# Assessment of arbuscular mycorrhizal fungi on the phytoremediation potential of *Ipomoea aquatica* on cadmium uptake

**DOI:** 10.1007/s13205-012-0046-8

**Published:** 2012-02-16

**Authors:** Anwesha M. Bhaduri, M. H. Fulekar

**Affiliations:** Environmental Biotechnology Laboratory, Department of Life Sciences, University of Mumbai, Santacruz (E), Mumbai, 400098 India

**Keywords:** Phytoremediation, Cadmium, AMF, *Ipomoea aquatica*, Antioxidant enzymes

## Abstract

The phytoremedial potential of *Ipomoea aquatica* and role of arbuscular mycorrhizal fungi (AMF) during Cadmium uptake was studied under two different soils i.e., soil inoculated with and without AMF. The plants were treated with different concentrations of Cd(NO)_3_ starting from 0, 5, 10, 25, 50, and 100 ppm in three replicate design in soil with and without AMF inoculation. Results showed that AMF enhanced accumulation of cadmium in plant tissues at all concentrations. Plants in AMF exhibited tolerance for Cd up to 100 mg/l and accumulated 88.07% in its tissues with no visual symptoms of toxicity, whereas those in non-AMF showed marked growth reduction at the same concentration with a metal accumulation of 73.2%. A significant variation of antioxidant enzymes under different environments evaluated the defense pathways of plants during uptake of Cd. Physiological changes and nutrient uptake showed that plants inoculated in AMF were more unwavering during stress conditions. The study established that phytoremedial potential of *I*. *aquatica* depends on rhizospheric conditions which enhanced Cd uptake. Finally, it was established that AMF was able to maintain an efficient symbiosis with *I*. *aquatica* in soil moderately contaminated by Cd, viable due to relation between fungus and plant.

## Introduction

Heavy metals like As, Cd, Co, Cu, Ni, Zn, and Cr are phytotoxic either at all concentrations or above certain threshold levels. They damage the environment by affecting soil fertility, biomass and crop yields and ultimately human health (Mudgal et al. 2010). Among the pollution producing metals, Cd is a widespread heavy metal in the environment and is regarded as non-essential elements and have half-life which is extremely persistent in the environment (Salt et al. 1998). Moreover, this toxic metal is also easily taken up by plants which in turn magnifies through food chain. Soil and water contaminated with such heavy metals pose a major environmental and human health problem that needs an effective and affordable technological solution. (Shu et al. 2003).

Phytoremediation is an emerging approach that offers ecological benefits and a cost efficient alternative to earlier remediation methods. It involves use of plants to partially or substantially remediate selected contaminants in contaminated soil, sludge, sediment, ground water, surface water, and waste water (Vishnoi and Srivastava 2008). Although it is a comparatively cheaper method, it requires technical strategy, expert project designers with field experience to choose the proper species and cultivars for particular metals, and utilizes a variety of plant’s biological processes and physical characteristics to aid in the site remediation (Mudgal et al. 2010).

Considering soil contamination with Cd and need for its remediation, researchers have tried to find plant species which have the capability of accumulating Cd (Kashem et al. 2008). Macrophytes have been used during the last two decades for metal removal (Denny and Wilkins 1987). One such macrophyte *Ipomoea aquatica* is a semi-aquatic tropical plant grown as a leaf vegetable. In the present study this plant was selected due to its easy establishment in terrestrial conditions, tolerance, and growing easiness.

The study on effect of Cd on growth, physiological, and biochemical processes of plants can in turn have the potential to bring new solutions for remediation of heavy metals contamination. These changes can be best evaluated when the same plant is subjected to different environmental conditions. A comparative study of response of a plant in soil inoculated with arbuscular mycorrhizal fungi (AMF) and non-AMF was adopted to investigate the sequence of some physiological and biochemical changes and factors that may interfere with tolerance mechanism of plants during uptake of heavy metals. Arbuscular mycorrhizal fungi are ubiquitous soil microorganisms and obligate symbionts, which confer a direct link between soil and roots, enhancing plant mineral nutrition, water acquisition, and resistance to biotic and abiotic stresses. (Harrier and Watson 2003).

Therefore, the aim of present study was to evaluate the role of AMF in phytoremediation of Cd by *I*. *aquatica*.

## Materials and methods

### Collection of aquatic plants

Plants were collected from a fresh water pond in University of Mumbai, Kalina Campus, Mumbai India. Collected plants were washed with deionized water and grown in Hoagland nutrient solution (Hoagland and Arnon 1950) to select freshly grown plants with developed roots of same biomass for the study. The selected plants were transferred to soil with and without AMF (Source: Dubey and Fulekar 2011) for a period of 28 days in 1 kg of soil respectively. Different concentrations of Cd(NO)_3_ (SRL, Mumbai) used in the study were 5, 10, 25, 50, and 100 ppm. Plants transferred to soil without metals served as control. Each treatment was carried out in triplicate design. The sampling of soil containing metals were done on day 0, 1, 3, 7, 21, and 28 respectively for evaluating Cd depletion from media and parameters that could interfere with uptakes, such as change in nutrient content, CFU. At the end of the experiment the plant samples were collected and washed with deionized water twice and rinsed with distilled water for different studies.

### Determination of metal uptake by plants

Plants removed from the solution were washed thoroughly in distilled water. All samples were air dried followed by oven drying at 60 °C for 72 h and digested in a mixture (5:1) ratio of concentrated nitric acid and perchloric acid (Thomas Becker, Mumbai) on a hot plate at a constant temperature of 70–100 °C.

The digestion process was continued until a clear solution remained after volatilization of acids, and was stopped when the residue in the flask was clear and white. The digested sample was dissolved in distilled water, filtered to remove impurities (APHA et al. 1998) and made up to the desired volume. The digested samples were subjected to analysis of the metals by atomic absorption spectrophotometer (AAnalyst 800, Perkin Elmer, USA) using flame atomization. The results are expressed on dry weight basis of each component. The metal content in control soil was not detectable and hence taken as 0 ppm.

### Determination of enzyme activity

Activities of catalase (CAT), superoxide dismutase (SOD), ascorbate peroxidase (APX) and guaiacol peroxidase (GPX) were determined spectrophotometrically. Catalase activity was evaluated by the decomposition of H_2_O_2_ followed by a decline in absorption at 240 nm (Aebi 1983).

Superoxide dismutase activity was assayed by determining the inhibition rate of nitroblue tetrazolium reduction (Misra and Fridovich 1972), APX activity by oxidation of ascorbate to dehydroascorbate (Asada and Chen 1989). Guaiacol peroxidase activity was determined following the oxidation of Guaiacol (Upadhaya et al. 1985).

## Bacteria and fungus CFU count

### Microbial and fungal characterization

Microbial number was assessed after 28 days from AMF and non-AMF soil. Bacterial and fungal colony-forming units (CFU) were counted using the standard dilution plate technique of fresh soil suspension on selective media. Bacteria were determined on nutrient agar (Hi Media, Mumbai). Fungi were estimated on rose Bengal agar (Hi media). The results are expressed as logs of bacterial and fungal CFU per gram of dry soil (Dubey and Fulekar 2011).

$$ \log_{10} {\text{Reduction}} = \log_{10} \;{\text{Control\;(mean)}} - \log_{10} {\text{Sample}}\; ( {\text{mean)}} $$

### Statistical analyses

The results are expressed as arithmetic means (*n* = 3) of three replicates. Significant differences of measured parameters between AMF, non-AMF were determined by one way ANOVA at *p* < 0.05 and *p* < 0.1.

## Results

**Fig. 1:**
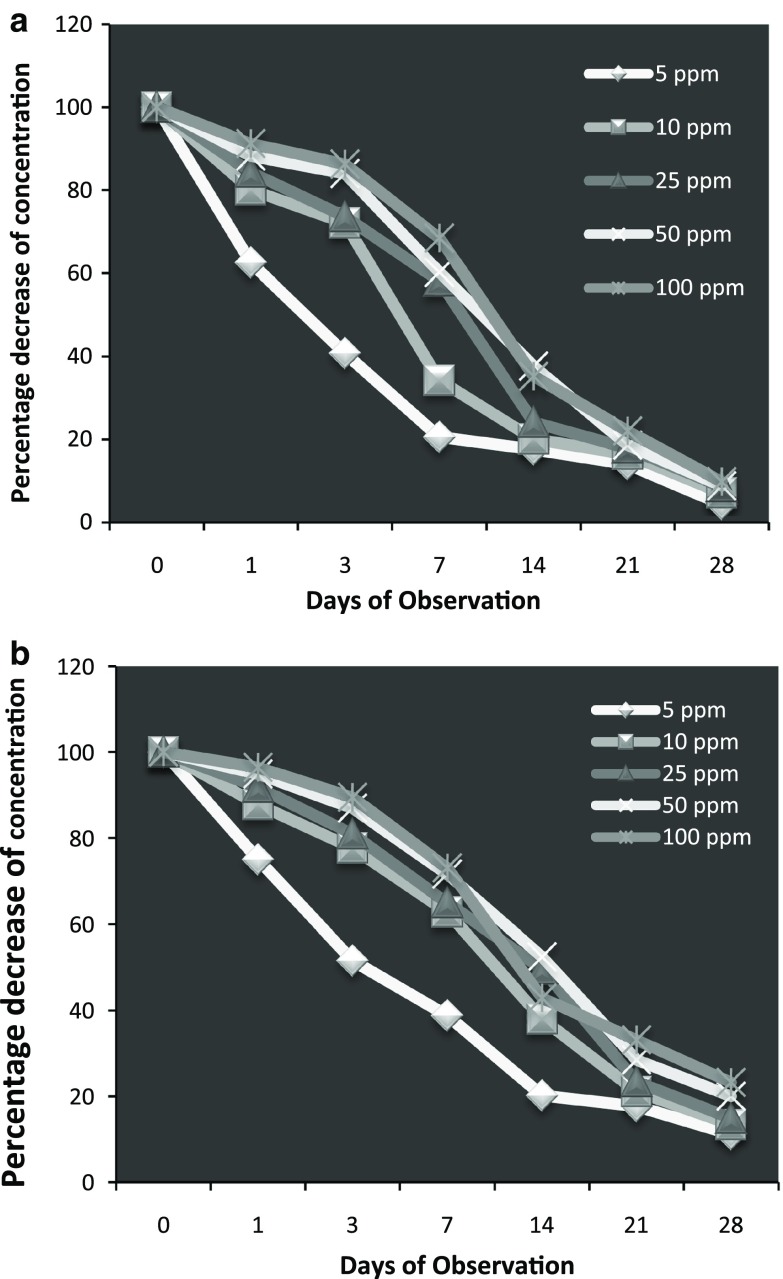
Percent depletion of Cd from different media (**a** AMF, **b** non-AMF) from day 0 to 28 days

The result presented in (Fig. 1) showed the uptake of heavy metals by *I*. *aquatica* from soils 28 days. The metal uptake by plants were directly proportional to the Cd concentration in soil. Cd was accumulated the highest at 100 ppm, i.e., 88.07, and 73.2% for AMF and non-AMF, respectively. At all concentrations the metal was accumulated more in roots than in stem in both conditions, i.e., more than 60% of metal was accumulated in roots in non-AMF and more than 78% in AMF inoculated soil. This is in accordance with results from (Kashem et al. 2008), that in *I*. *aquatica*, most of the absorbed Cd (>88%) get accumulated in the roots. The highest proportion of Cd in the roots of *I*. *aquatica* may be due to immobilization of Cd through precipitation and/or adsorption on the root surface and within the symplasm of root cells as well as due to sequestration of Cd by phytochelatins in the vacuoles of root cells (Shute and Macfie 2006). The uptake of Cd by plants in the present study clearly suggested that *I*. *aquatica* performed better in AMF as compared to non-AMF under moderately high concentration of Cd. Similar observation of significant effect of AMF and non-AMF have been also reported by Yu et al. (2004). The tolerance mechanisms of plants can be attributed to the role of AMF giving protection in heavy metal stress.

Reduction in the fresh and dry weights of root and shoot, leaf area, and leaf are among the most sensitive responses to Cd exposure and are the indices for stress responses like other physiological reactions (Hatata and Abdel-Aal 2008).

**Fig. 2:**
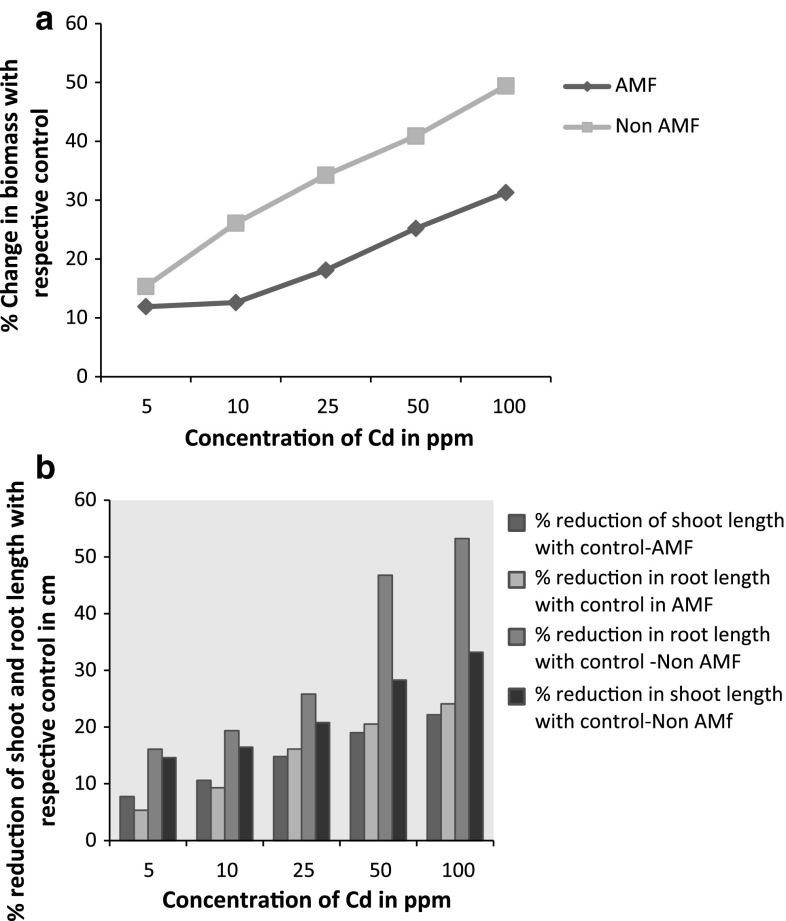
**a** Relative percent change of biomass at various concentrations with respective control of AMF and non-AMF. **b** Relative percent reduction of root and shoot length at various concentrations with respective control of AMF and non-AMF

Growth parameters in terms of total biomass, and root and shoot length were significantly (*p* < 0.05), (*p* < 0.1) affected by Cd concentration in both the conditions. In broad-spectrum AMF association exhibited higher growth in terms of fresh biomass, and root and shoot length (Fig. 2a, b). Moreover, plants removed from AMF did not show any visible symptoms of toxicity while roots turned yellow after the period of 28 days in non-AMF. Relative percent change with control in biomass at the highest concentration was 31.3% and 49.4% in AMF and non-AMF respectively (Fig. 2a). In both the cases, the drastic change in biomass were seen from 10 ppm where percent change with control were 12.6% and 26.1% for AMF and non-AMF, respectively. Therefore, AMF plants exhibited better biomass yield in toxic condition with no visible symptoms of toxicity as compared to non-AMF. Most reports note a positive effect of AMF inoculation on the growth of plants in metal-contaminated soils. This protective benefit may be related to the adsorptive or binding capability for metals of the relatively large fungal biomass associated with the host plant roots, which may physically minimize or exclude the entry of metals into host plants (Meharg and Cairney 2000).

From the study, it can be inferred that AMF helped in improving the nutrient availability for the plants. Phosphorous showed a significant (*p* < 0.05) amount in AMF plants as compared to non-AMF. There were also contributions from other nutrients like nitrogen and potassium during the uptake. Phosphorous and potassium were significantly (*p* < 0.05) high in AMF in all concentration as compared to non-AMF. Moreover, nitrogen content in plants was significantly (*p* < 0.1) higher in AMF as compared non-AMF. Although fungal association with plants enhanced nutrient uptake which in turn plays an important role in conferring plant tolerance to heavy metal stress, but it can be clearly depicted from our study that Cd addition had an injurious effect on plants since the uptake of nutrient irrespective of environmental conditions decreased considerably with increase in concentration, which is in accordance with study reported by Hatata and Abdel-Aal (2008) in sunflower. The enhanced nutrient supply, mainly phosphorus to the host plant by the AMF may attenuate the effect of physiological stress caused by Cd (Meharg and Cairney 2000).

**Fig. 3:**
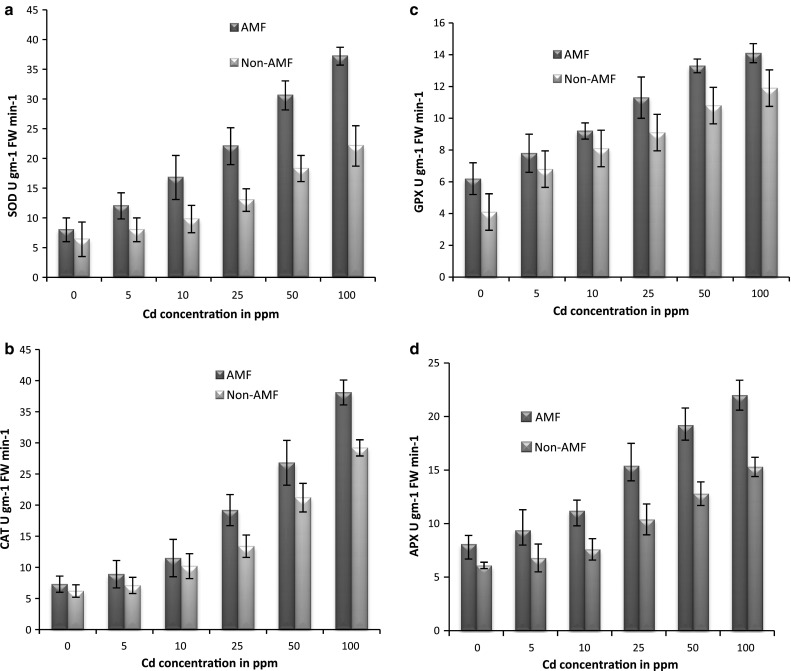
Antioxidant enzymes (**a** SOD, **b** CAT, **c** GPX, and **d** APX) response due to Cd-induced toxicity in plants in AMF and non-AMF soil

Taken together the results of all antioxidant enzymes which showed quite a similar variation with increasing concentrations in both AMF and non-AMF plants. Hence it can be concluded that antioxidant activity was induced as a tolerance mechanism against the toxic effects of Cd. Although variations of plant enzymes expressed similar relations in both the conditions, AMF showed significantly (*p* < 0.05) higher enzyme activity than non-AMF with increasing concentrations (Fig. 3). It is possible that roots in mycorrhizal associations make available powerful physiological defense against Cd to cope with toxicity. Plants in AMF showed consistent increase in antioxidant enzymes with increase in concentration and better growth rate as compared to non-AMF as possible mechanisms for plant protection against high accumulated toxic heavy metals in shoots (Tong et al. 2004).

**Fig. 4:**
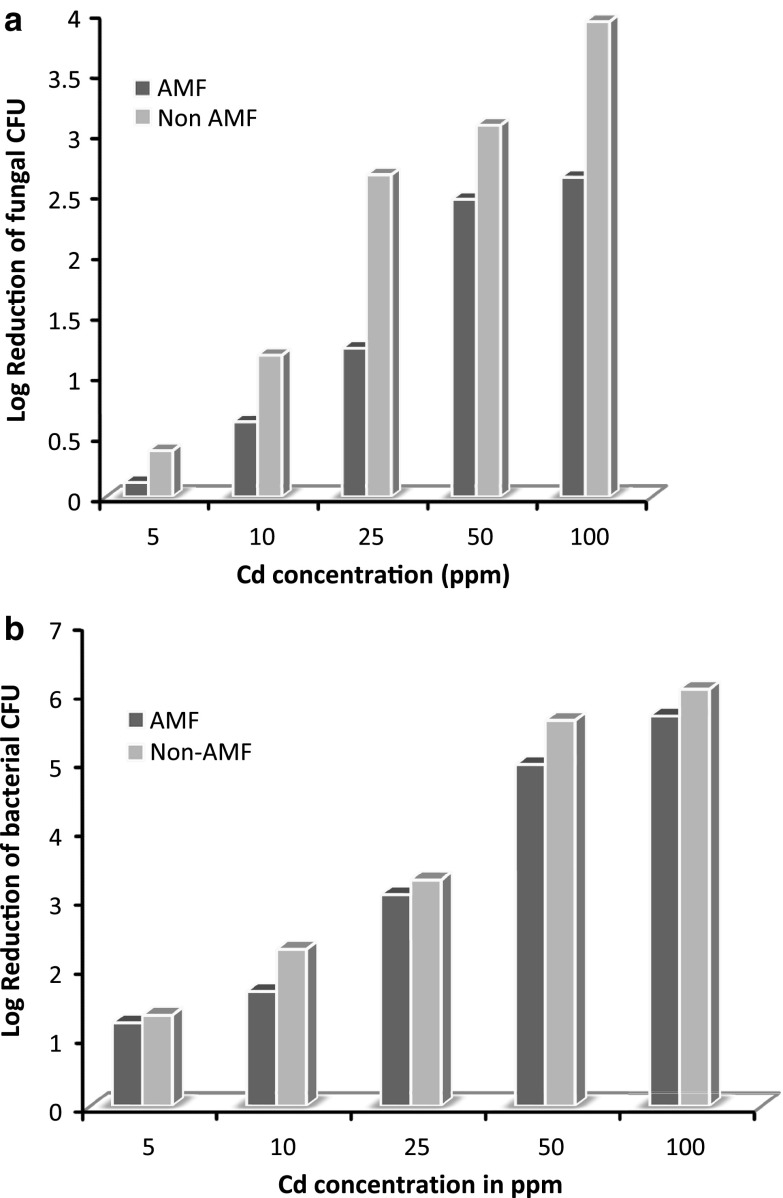
Log reduction of bacterial and fungal CFU in AMF and non-AMF (log CFU/gm of soil)

The results in (Fig. 4) clearly show that soil microorganisms played a major role in combating toxic effect of Cd. It can be accomplished that efficient mycorrhizae symbiosis with *I*. *aquatica* promoted plant efficiency for uptake of Cd. Therefore availability of heavy metals to plants and their toxicity depends on complex rhizospheric reactions involving not only exchange processes between soil and plants but also microbial activities (Schützendübel et al. 2001). It is seen that microbial count reduction in AMF due to Cd toxicity was lesser than non-AMF. Hence rhizospheric interactions in AMF seemed to help in nurturing the bacteria in toxic conditions or may be the nutrient availability help them cope up the toxic effect of Cd. Thus, metal uptake and tolerance depend on plant, soil, and/or the environment including microbes and their interaction with plant roots and their symbionts like AM fungi.

## Conclusion

The present study shows that plants used for phytoremediation provide basic understanding of the numerous issues like the tolerance mechanism of plants during stress, role of microorganisms and nutrient availability for a successful symbiosis between plant and the environment. *Ipomoea aquatica* under the above conditions can remediate and tolerate moderate contamination of cadmium in soil. The association between microbes and plant and other factors such as AMF species, metal ion type should be taken in consideration for a successful phytoremediation. Moreover, as observed during the study plants did not show any symptoms of toxicity in AMF till 100 ppm which opens us with the opportunity to carry out the study by further increasing the concentrations of the metals.
